# Novel coronavirus pandemic: A clinical overview

**DOI:** 10.4102/safp.v62i1.5123

**Published:** 2020-06-26

**Authors:** Ramprakash Kaswa, Indiran Govender

**Affiliations:** 1 Department of Family Medicine and Rural Health, Walter Sisulu University, Mthatha, South Africa; 2 Department of Family Medicine, Kalafong Hospital, Pretoria, South Africa; 3 Department of Family Medicine, University of Pretoria, Pretoria, South Africa

**Keywords:** COVID-19, SARS-CoV-2, coronavirus, pneumonia, PPI, IPC

## Abstract

The outbreak of severe acute respiratory syndrome coronavirus 2 (SARS-CoV-2) is an emergent public health crisis threatening the current world health establishment. The SARS-Co-2 was first identified in Wuhan, Hubei Province, China, in December 2019. There have been about 6.5 million reported cases of coronavirus disease 2019 (COVID-19) and about 350 000 reported deaths throughout the world within the last 6 months from the onset of the epidemic. The virus is primarily transmitted by inhalation or contact with infected droplets. The COVID-19 patient usually presents with fever, cough, sore throat and breathlessness. Currently, available data indicate that the majority of people with the disease have mild symptoms, while about 20% present with moderate-to-severe disease. About 5% of these may progress to pneumonia, acute respiratory distress syndrome and multi-organ dysfunction. To date, there is no recommended medical treatment, and supportive measures are a crucial part of management. The case fatality rate of SARS-CoV-2 is lower than that of its two coronavirus predecessors, that is, severe acute respiratory syndrome coronavirus (SARS-CoV) and Middle East respiratory syndrome coronavirus (MERS-CoV). The full impact of this new pandemic on health, social and economic well-being of humankind is yet to be ascertained.

## Background

On 31 December 2019, a cluster of pneumonias of unknown aetiology was reported to the World Health Organization (WHO) from Wuhan City, Hubei Province, China. A week later, a novel coronavirus was identified by the Chinese Centre for Disease Control and Prevention (CDC).^1,2^ Subsequently, the causative agent was named 2019-nCoV by the WHO.^3^ Coronaviruses are a large family of viruses that can cause asymptomatic infection, or symptoms ranging from mild flu-like illness to severe pneumonia.^4,5,6^ Middle East respiratory syndrome (MERS-CoV) and severe acute respiratory syndrome (SARS-CoV) are common examples of severe respiratory tract infections caused by coronavirus in humans.^7^

Taxonomically, coronaviruses are members of the subfamily Orthocoronavirinae in the family Coronaviridae and the order Nidovirales. This subfamily Orthocoronavirinae includes four genera: *Alphacoronavirus, Betacoronavirus, Gammacoronavirus* and *Deltacoronavirus*. The *alpha coronaviruses* and *beta coronaviruses* infect mammals only. The *gamma coronaviruses* and *delta coronaviruses* mainly infect birds, but some of them can also infect mammals.^7,8^ Coronaviruses are categorised according to the severity of disease they cause in humans, as follows:

- Lower pathogenicity^7^:
  ■ HCoV-229E (alpha coronavirus)
  ■ HCoV-NL63 (alpha coronavirus)
  ■ HCoV-OC43 (beta coronavirus)
  ■ HCoV-HKU1 (beta coronavirus)
- Higher pathogenicity^7^:
  ■ SARS-CoV (beta coronavirus)
  ■ MERS-CoV (beta coronavirus)
  ■ SARS-CoV-2 (beta coronavirus)

This new strain of the novel coronavirus (nCoV) was identified for the first time in humans, and the sequence of the virus is phylogenetically similar to the other six coronavirus subtypes.^7^ The genomic sequence of this new strain, classified as *betacoronavirus*, is close to SARS-CoV and was subsequently named SARS-CoV-2. It is an enveloped virion and has a single positive-sense ribonucleic acid genome. It measures approximately 50 nm – 200 nm in diameter. The club-shaped glycoprotein spikes on the envelope give the virus a crown-like or coronal appearance.^9^

All seven human coronaviruses are zoonotic, meaning that they are transmitted from animals to humans.^7^ The MERS-CoV was transmitted from dromedary camels to humans and SARS-CoV from civet cats to humans. Several other known coronaviruses are circulating in animals, but most of them have not yet infected humans.^7,10^

The major outbreaks of coronaviruses were SARS-CoV during 2002–2003 and MERS-C0V during 2012. The SARS-CoV outbreak involved 8422 patients and spread to 29 countries globally, with a case fatality rate of 9.6% during 2002–2003.^11^ Middle East respiratory syndrome emerged in Middle Eastern countries and infected 2494 patients, with a case fatality rate of 34% during 2012.^4^ The case fatality rate of SARS-CoV-2 is reported to be from 0.5% to 3.5%, compared to a seasonal flu where case fatality rate is 0.1%. On the basis of the current evidence, the mortality rate of COVID-19 is five to 35 times higher than the normal seasonal influenza.^1,6,12^

## Clinical presentation of coronavirus disease 2019

**FIGURE 1:**
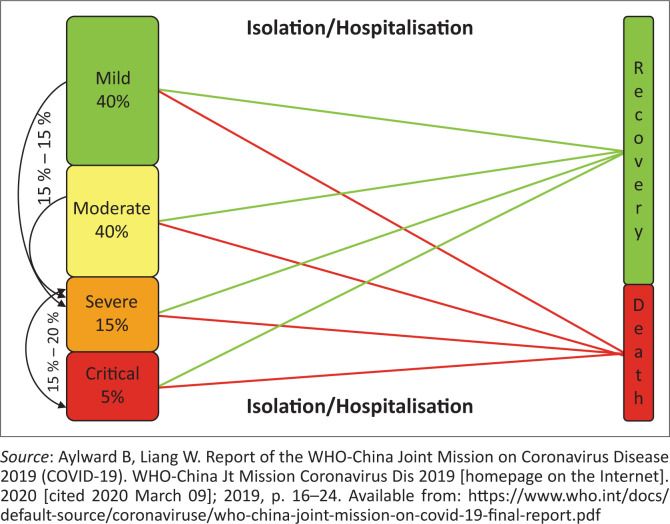
Spectrum of clinical presentation and progression of severe acute respiratory syndrome coronavirus 2.

The presentation of COVID-19 ranges from mild, self-limiting respiratory tract infection to progressive severe pneumonia, leading to death. According to the current evidence, 80% of patients develop only mild symptoms, an estimated 15% develop severe illness with hypoxaemia and about 5% become critically ill with respiratory failure (Figure 1).^11,13,14^ Fever is the most common presenting symptom but is not present in all cases. Other common symptoms include cough, fatigue, sore throat and headache. The common signs include shortness of breath, myalgia or arthralgia and chills. An atypical presentation could be nausea or vomiting and diarrhoea.^2,14,15^

**FIGURE 2:**
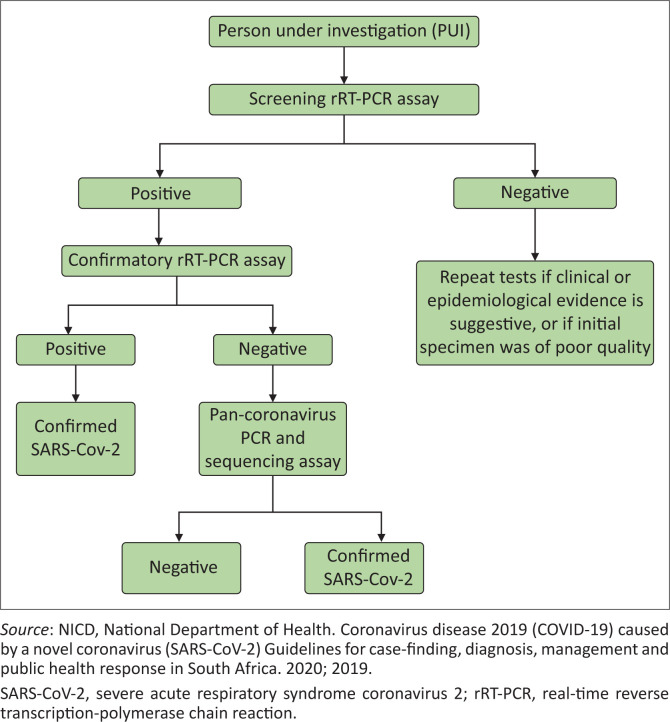
Algorithm for testing person under investigation for coronavirus disease 2019.

The severity of the disease in patients is classified according to the following criteria^14^:

- shortness of breath
- respiratory rate > 30 breaths per minute in an adult
- SpO2 ≤ 95
- chest X-ray with multi-lobar infiltrates or pulmonary infiltration progressing to > 50% within 24 h – 48 h.

People with advanced age, diabetes, human immunodeficiency virus (HIV) infection or long-term use of immunosuppressive agents, and those with comorbidities, are associated with higher mortality.^1,16,17,18^

Case definition of coronaviruses disease 2019^12^:

- The case definition of COVID-19 is persons presenting with a sudden onset of acute respiratory illness and at least one of the following symptoms: cough, shortness of breath, sore throat and fever (≥ 38 °C), or a history of fever, irrespective of admission status.
- Persons who have an acute respiratory illness and in the 14 days prior to the onset of symptoms met one of the following epidemiological criteria are at the highest risk^2,19^:
  ■ In close contact with a confirmed or probable case of SARS-CoV-2 infection.
  ■ History of travel to areas with local transmission of SARS-CoV-2.
  ■ Worked in, or attended a healthcare facility where patients with SARS-CoV-2 infections were being treated.
  ■ Admitted with severe pneumonia of unknown aetiology.

### Transmission^3^

Person-to-person transmission of SARS-CoV-2 is common. Personal contact and respiratory droplets are the main routes of transmission.^3^ The virus is also excreted in stool but so far no case of faeco–oral transmission has been reported. No mother-to-child transmission (MTCT) of SARS-CoV-2 has been reported during the first 6 months of the outbreak.^20^ The current research has demonstrated that the virus can also transmit by coming in contact with a contaminated surface, and viral stability on a surface depends on relative temperature, humidity and types of surface materials. The estimated mean incubation period for COVID-19 is 4–5 days, with a range of 2–7 days.^9,12,17^ The viral reproductive number (R_º_) is about 2.2.^14^

Although WHO has identified symptomatic cases as the main driver of transmission of SARS-CoV-2, The possibility of transmission prior to developing symptoms is a matter of grave concern, but it remains to be defined.^2,3^ Viral shedding in SARS-CoV-2 is the highest early in the course of disease, compared to other coronaviruses where peak shedding occurs around 5 days after the onset of symptoms. In SARS-CoV-2, viral shedding can occur even 12 h – 48 h prior to the onset of symptoms.^14^ Viral shedding is continuous from 7 to 12 days in mild to moderate cases, and in severe cases it continues beyond 2 weeks.^3,4,10^

### Isolation criteria

**FIGURE 3:**
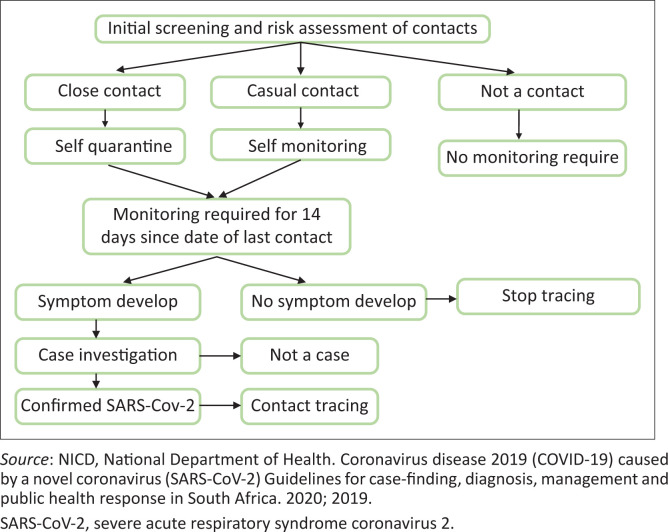
Flow diagram for contact tracing, screening and monitoring of coronavirus disease 2019.

Isolation criteria may be applied in different ways during the course of the coronavirus epidemic. The following criteria are currently applied for hospitalised patients in South Africa (Figure 3):

- Mild cases can be isolated for 14 days after the onset of symptoms while in moderate-to-severe cases, after achieving clinical stability, the patient should be isolated for 14 days.^3,4^ The period of viral shedding from the upper respiratory tract is shorter in mild cases than in severe cases.
- In severe disease, viral shedding can be continuous for a longer time period, and patients should be isolated for 14 days after supplementary oxygen has been discontinued.^4,12^

The approximate duration of viral shedding of SARS-CoV-2 is 20 days, with a range of 8–37 days. Although asymptomatic patients have viral loads similar to those of symptomatic patients, they are less likely to be infectious.^14^ Patients who remain asymptomatic after testing positive for SARS-CoV-2 should be isolated for 14 days from their first positive test.^12,17,18^ There is no need for a retest at the end of the isolation period.^12^

### Personal protective equipment

All healthcare workers who are involved in the management of confirmed cases of COVID-19 must use appropriate personal protective equipment (PPE), consisting of gloves, apron or gown and a surgical mask. When health care workers perform aerosol-generating procedures on a suspected or confirmed COVID-19 patient, they should use an N95 respirator, gloves, apron or gown and eye protection (shield or goggles).^3^ Taking nasopharyngeal/oropharyngeal swabs, bronchoscopy, open suctioning of the respiratory tract, performing cardiopulmonary resuscitation (CPR) and intubating a COVID-19 patient are regarded as aerosol-generating procedures.^4,8^

### Infection prevention and control

Infection prevention and control (IPC) is an integral part of the management of COVID-19 patients.^1,18^ *Standard precautions should always be applied to all patients in health care facilities, irrespective of their diagnosis*. These precautions include health care personnel hand hygiene, disposal of sharps, health care waste management, disinfection of patient care articles, respiratory hygiene, appropriate use of PPE, and occupational health and injection safety.^4,11,17^

### Laboratory tests

The reverse transcriptase polymerase chain reaction (RT-PCR) test is currently available for SARS-CoV-2 infection in South Africa (Figure 2), but in future other tests, including antibody and antigen detection assay, could be part of laboratory dignosis.^12^ Influenza is the most common *differential diagnosis* of suspected cases of COVID-19. Atypical and conventional bacterial pneumonias, and *pneumocystis jirovecii* pneumonia (PJP) in patients with HIV should be considered as other differentials.^8,11,12^ Depending on the clinical presentation of a patient, the following samples may be needed to guide management:

- full blood count and differential count
- blood cultures
- nasopharyngeal and oropharyngeal swabs for viral and atypical pathogens
- chest X-ray
- sputum for microscopy, culture and sensitivity (MC&S)
- GeneXpert mycobacterium tuberculosis/resistance to rifampicin (MTB/RIF) Ultra
- urine for lipoarabinomannan (LAM) test, if HIV-positive.

The diagnosis of conventional respiratory pathogen does not rule out SARS-CoV-2 infection.^12^

Common complications of coronavirus disease 2019^15^:

- Overall, pneumonia is the most common complication in COVID-19 patients.
- Among critically ill patients, the following complications are reported:
- acute respiratory distress syndrome (ARDS)
- shock or septic shock
- acute kidney injury/renal failure
- acute hepatic injury
- cardiac abnormalities, for example, acute cardiac injury, cardiomyopathy or arrhythmia
- hospital-acquired infection/ventilator-associated pneumonia.

Common laboratory findings reported in coronavirus disease 2019^15^:

- lymphopenia
- thrombocytopenia
- leukopenia
- elevated aspartate transaminase (AST), alanine transaminase (ALT) and much higher with severe disease
- procalcitonin, typically normal on admission
- increased lactate dehydrogenase, C-reactive protein (CRP) and serum levels of pro-inflammatory cytokines and chemokines
- increased D-dimers.

### Management of coronavirus disease 2019 contact

A contact is a person who fulfils the following criteria:

- a person in direct care or staying in the same environment as that of a COVID-19 patient
- working with healthcare workers infected with a COVID-19 patient
- working together or having close proximity to a COVID-19 patient
- travelling with a COVID-19 patient in any kind of conveyance
- sharing the same household with a COVID-19 patient.

Persons who have been exposed to a suspected or confirmed COVID-19 patient need to isolate themselves and monitor their health for 14 days from the last day of possible contact.^3^

### Clinical management of coronavirus disease 20-19

The goal in clinical management of cases is to reduce morbidity and mortality, and minimise transmission to uninfected contacts. This means triaging patients, and early recognition of hospital or intensive care unit admission will be essential for reducing morbidity and mortality.^15,17^ Implementation of IPC measures and contact tracing is crucial for minimising onward transmission of the virus.^9^

### Mild disease

Patients with stable mental status, SpO2 ≥ 95%, respiratory rate < 25, heart rate (HR) < 120 and temperature 36°C – 39 °C are considered to have mild disease.^16,18^ All patients with mild disease who are aged less than 65 years and have no cardiac or pulmonary comorbidities may be considered for constant monitoring and management at home.

### Moderate and severe disease

Oxygen therapy with target SpO2 ≥ 92% – 95% is likely to be the single most effective supportive measure in COVID-19 patients. All patients who develop ARDS need lung-protective ventilation strategies.^11,15,20^

### Empiric treatment of other pathogens

Where the patient has confirmed other pathogens, consider the following empiric treatment:^11,12^

- amoxicillin-clavulanate for community-acquired pneumonia pathogens
- azithromycin for atypical pneumonia pathogens
- Influenza management guidelines for severe influenza
- co-trimoxazole for PJP.

To date, no specific treatment for COVID-19 has been found, but the following drugs are under investigation for inpatient clinical management:^13,16^

- hydroxychloroquine or chloroquine^16^
- lopinavir/ritonavir
- remdesivir
- IL-6 blockers.

## Prognosis of coronavirus disease 2019

The majority of cases recover fully with supportive care, although this may take several weeks. A minority of cases, particularly severe cases, progress to ARDS, multiple organ failure and sometimes even death.^1,11,12^ Increased D-dimers and lymphopenia in laboratory findings are associated with high mortality.^10,15^

## Conclusion

This novel virus outbreak has challenged the public health infrastructure, and time alone will tell how the global emergence of this virus will impact our daily lives. COVID-19 is a highly contagious disease, and to prevent its community spread, primary care physicians have to have a high index of suspicion in patients presenting with respiratory symptoms. At the time of this writing, there is no approved treatment and vaccine. Infection prevention and control measure is an integral part of COVID-19 management. Primary health care providers must make every effort to curb this outbreak.
